# Comparison of Resilience, Personal Recovery, and Quality of Life Measures Pre- and Post-Discharge from Inpatient Mental Health Units in Alberta: Analysis of Control Group Data from a Randomized Trial

**DOI:** 10.3390/healthcare11222958

**Published:** 2023-11-14

**Authors:** Ernest Owusu, Reham Shalaby, Hossam Elgendy, Wanying Mao, Nermin Shalaby, Belinda Agyapong, Angel Nichols, Ejemai Eboreime, Nnamdi Nkire, Mobolaji A. Lawal, Vincent I. O. Agyapong

**Affiliations:** 1 Department of Psychiatry, University of Alberta, Edmonton, AB T6G 2R3, Canada; 2 Queen Elizabeth II Hospital, Alberta Health Services, Grande Prairie, AB T5J 3E4, Canada; 3 Department of Psychiatry, Dalhousie University, Halifax, NS B3H 4R2, Canada

**Keywords:** hope, hospital discharge, mental health, recovery, resilience, transition

## Abstract

**Background:** The transition from hospital to community settings for most mental health service users is often hindered by challenges that affect community adjustment and continuity of care. The first few weeks and days after discharge from mental health inpatient units represent a critical phase for many service users. This paper aims to evaluate the changes in the resilience, personal recovery, and quality of life status of individuals with mental health challenges recently discharged from acute mental health care into the community. **Methods:** Data for this study were collected as part of a pragmatic stepped-wedge cluster-randomized, longitudinal approach in Alberta. A paired sample *t*-test and Chi-squared/Fisher test were deployed to assess changes from baseline to six weeks in the recovery assessment scale (RAS), brief resilience scale (BRS), and EuroQol-5d (EQ-5D), using an online questionnaire. **Results:** A total of 306 service users were recruited and 88 completed both baseline and six weeks, giving a response rate of 28.8%. There was no statistically significant change in the level of resilience, recovery and quality of life as measured with the brief resilience scale, recovery assessment scale and EQ-5D from baseline to six weeks (*p* > 0.05). **Conclusions:** The study showed that there was neither an improvement nor deterioration in resilience, recovery, or quality of life status of service users six weeks post-discharge from inpatient mental health care. The lack of further progress calls into question whether the support available in the community when patient’s leave inpatient care is adequate to promote full recovery. The results justify investigations into the effectiveness of innovative and cost-effective programs such as peer and text-based supportive interventions for service users discharged from inpatient psychiatric care.

## 1. Background

The discharge process from acute mental health inpatient care has been described as incredibly stressful, and re-adaptation into the community as overwhelming. Both tend to increase the risk of subsequent rehospitalization [1,2,3]. The transition from hospital to society for most mental health service users is often hindered by challenges that affect community adjustment and continuity of care [4]. The first few days and weeks after discharge from mental health inpatient units represent a critical phase for many service users. Planning discharge services for persons with mental health issues involves stakeholders with different roles and expectations regarding the type of information required and the necessary level of involvement of people with mental health issues [5]. 

During this crucial phase of transition into the community, challenges may arise, such as anxiety, increased risk of suicide, craving, loneliness, lack of self-esteem, stigmatization, adherence to treatment challenges, and difficulties in dealing with recurring symptoms [6,7,8]. Other areas of concern are the service user’s ability to cope and maintain daily activities such as bathing, dressing, mobility, and general well-being [9]. The service user’s ability to develop resilience to deal with these is also critical. Their inability to cope may negatively impact their mental health, increasing the risk of growing conditions such as depression and anxiety [10]. Therefore, it is necessary, as part of the discharge planning process, for service users to acknowledge their responsibility for managing their recovery. This will prepare them to face life outside the hospital with some hope [11,12]. Assisting these persons to be in control may reduce their stress in having to do many things simultaneously in the community [13,14]. Moreover, service users can achieve a successful personal recovery [15] since there is evidence to prove that service users with mental health challenges can return to fulfilling lives with appropriate support and compliance to treatment [16].

After discharge, persons with mental health challenges identified unmet needs related to their condition in the community. Also, some family members of the service users expressed concerns about the lack of improvement in the mental health of their relatives over time [17,18]. The inability of most service users to ask for help can affect their level of confidence and hope [19]. Furthermore, it is estimated that one-third of all suicides among service users with mental health challenges occur within the first three months following hospital discharge [20,21]. There is enough evidence to support the fact that mental health follow-up services can reduce the risk of suicide and readmission [22,23]. Rehospitalization also tends to increase if the transition to the community is not well coordinated and planned [3]. For example, in Alberta, the average wait time for most service users with mental health problems from the point of referral to the first appointment at an Addiction and Mental Health (AMH) unit was approximately 38 days and 28 days in Edmonton and the North Zones, respectively, in 2019 [24,25]. The long wait times unfortunately leave persons with mental health challenges unsupported, contributing to their conditions worsening and the resulting impact is their readmission into acute care [24]. It is also estimated that the long wait times and the high no-show rates result in approximately 10% of service users in Alberta relapsing and being readmitted within three months of discharge from acute care [26]. Readmission rates may reflect the quality of services and support at the community level. It may also reflect the inefficiency in the acute care systems [3,26]. Support at discharge is one of the tools to reduce the risk of readmission [26]. 

Prevention of readmission is likely the responsibility of the community care provider rather than the mental health hospital once the hospital has arranged an initial aftercare plan in collaboration with the community provider [27]. Another consequence or impact of the gap created during the transition to the community is the attendant need for specialty care which invariably leads to an increased financial burden on the healthcare provider [26,28]. Mental health challenges are the most prevalent disabilities in Canada, accounting for 70% of documented costs with a cumulative annual economic impact of approximately $8 billion in direct costs and between $11 billion and $50 billion in indirect costs [29]. The most prominent mental health problems in Canada are depression and bipolar disorder, with an estimated prevalence of 4.7 and 1.5%, respectively. It is also estimated that 11.3% and 2.6% of adults will report symptoms of major depressive and bipolar disorders, respectively, at some point in their lives [30]. Depression is a diverse condition and is approached with an equally diverse set of treatments, such as the use of medicine, counseling, peer support, recovery education, and other psychosocial interventions [31]. The report further indicated that less than 25% of Albertans with depression had their needs met psychologically, and it revealed disparities in the distribution of addiction and mental health services, with rural and northern communities being disadvantaged [24]. The evaluation of changes in the mental health of service users who have recently been discharged from acute mental health care into the community in several clinical and non-clinical domains is crucial. The domains are the level of resilience, quality of life in terms of the level of mobility, self-care, health status, level of pain and discomfort of the service users, and recovery in terms of the level of personal confidence and hope, goal, and success, the level of willingness to ask for help, the level of reliance on others and no domination by symptoms. 

The aim of this paper is to evaluate the changes in the resilience, personal recovery, and quality of life measures for service users recently discharged from inpatient mental health care into the community.

### Hypothesis

We hypothesize that:

1. The mean scores on the brief resilient scale (BRS), would be higher at six weeks post-discharge compared to baseline, suggesting improvement in their level of resilience.
2. The mean scores on the recovery assessment scale (RAS), would be higher at six weeks post-discharge compared to baseline, suggesting improvement in their level of recovery, six weeks after discharge from inpatient care.
3. Mean scores on the EQ-5D-5L subscales would be higher at six weeks post-discharge compared to baseline, suggesting improvement in their level of quality of life six weeks after discharge from inpatient care.

## 2. Methodology

### 2.1. Study Setting and Design

This study was conducted in the province of Alberta, Canada. Alberta has an estimated population of 4,695,290, according to Alberta population estimates released by the Government of Alberta on 1 July 2023. Six main acute mental health units across three main cities were the main sites, namely, Edmonton, Calgary, and Grand Prairie in Alberta [26]. The data in this study was collected as a part of a pragmatic stepped-wedge cluster-randomized, longitudinal approach employed to provide supportive text messages (Text4Support) and peer support services (PSS). Participants were recruited across six acute care sites across Alberta as clustered units of randomization [26]. The project was launched in March 2022, and service user recruitment started on 8 March 2022. The stepped wedge approach was deployed in four clusters over a period of 3 years, with interventions spread over quarters within a year. The researchers designed the program to evaluate two central innovative interventions, Text4Support and peer support, to reduce inpatient readmission rates for individuals discharged from acute mental health care [26].

### 2.2. Sample Size Calculation

With a projection that the effect size for the reduction in mean RAS, BRS, and EQ-5D scores at six weeks from baseline would be 0.5, a population variance of 1.0 for each scale mean score, a two-sided significance level of α = 0.05, and a power of 90% (β = 0.1) using an online script [32], we estimated that the sample size needed to assess the effects of the six-weeks transition from inpatient mental health care to community care on the outcome variables would be 44.

### 2.3. Ethics Statement

The Health Research Ethics Board of the University of Alberta (Ref # Pro00111459) provided the required ethical clearance for this study. Additional operational approval was obtained from the regional health authority. Signed written informed consent to access health records was obtained from all participants prior to inclusion into the study. Ethical approval was also obtained for verbal consent to interviews and implied consent for electronic survey responses.

### 2.4. Data Collection

**Figure 1:**
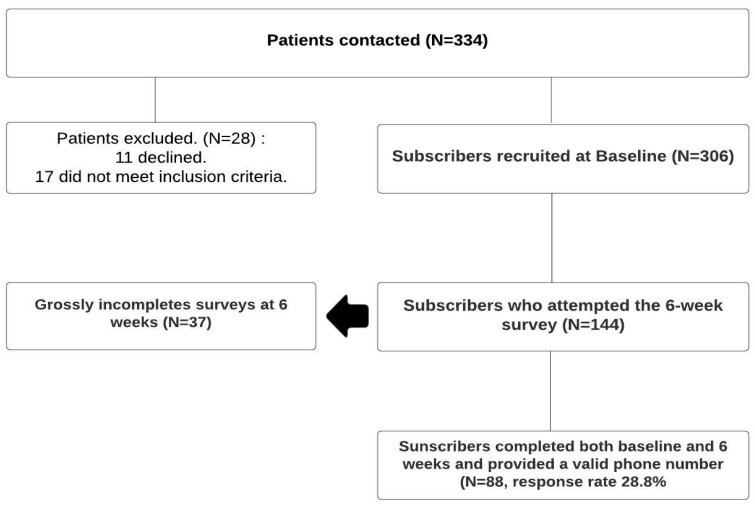
Study Flow Chart.

The data for this study was collected through REDCap [33] as part of a large ongoing trial assessing the utility of Text4Support and peer support in reducing inpatient readmission rates [26]. The eligibility criteria were a service user diagnosed with any mental condition, ready for discharge from an inpatient mental health unit, aged between 18 and 65, who had a mobile device, could read English text messages, and could provide informed written consent. Other essential data collected included sociodemographic information such as age, gender, ethnicity, educational level, relationship status, and employment status. Clinical information such as diagnosis and duration of the present admission of the service user were also collected. Recruitment for the randomized trial commenced in March 2022 and will be ongoing until March 2024. However, data for this sub-study was collected between 8 March 2022, and 31 May 2022, and all participants were in the control group of the randomized trial, receiving only the usual follow-up care post-discharge from the hospital. All study participants completed the baseline online survey with the assistance of a research team member after signing a paper-based consent form. They received a text message at six weeks post-discharge with a link to the follow-up survey. To maximize the response rate, a reminder text message was sent two days after the first follow-up text message was sent. Phone numbers were the primary identifier for the service users and were used to track the responses across the follow-up time points. Figure 1 represents the subscriber flowchart, which indicates the number of subscribers who completed the surveys at each time point of the data collection.

### 2.5. Outcome Measures

The primary outcome measures of interest included changes in the levels of resilience, recovery and quality of life as measured by mean scores on the brief resilience scale (BRS), the recovery assessment scale (RAS) [34,35] and the visual analogue score (VAS) on the EuroQol-5d-5L (EQ-5D-5L) [36]. The secondary outcome measures of interest included changes in resilience classification and categorical scores for sub-domains of the EQ-5D-5L from baseline to six-week post-discharge from inpatient psychiatric care. 

The BRS is a fast and simple self-assessment tool used to evaluate the level of resilience. The term resilience is the perceived power to bounce back or recover from stress [37]. The scale consists of six statements for which an individual can express to what extent they agree or disagree with them. This scale was developed to assess a unitary resilience construct, including positively and negatively worded items [37]. The scoring was by adding the responses varying from 1–5 on the Likert scale for all six items, ranging from 6–30. The total sum was divided by the total number of questions answered. When completed, it gave a resilience score of between 6 and 30, where higher scores indicated higher levels of resilience. The average score of 1.00–2.99 indicates low resilience, 3.00–4.30 normal resilience, and 4.31–5.00 indicates high resilience [38]. In this paper, we combined the normal and the high resilient levels in one category [3.00–5.0] against low resilience [1.00–2.99]. BRS has demonstrated good psychometric properties, including acceptable internal consistency reliability (*α* = 0.66) and test–retest reliability (*r* = 0.67) [39]. 

The RAS is a 24-item scale that provides self-reported recovery ratings on a 5-point Likert scale (strongly disagree = 1, disagree = 2, not sure = 3, agree = 4, and strongly agree = 5) [34,35]. The RAS subscales include five factors: (1) personal confidence and hope (response range 9–45); (2) willingness to ask for help (response range 4–20); (3) goal and success orientation (response range 3–15); (4) reliance on others (response range 5–25); and (5) no domination by symptoms (response 3–15) [40,41]. RAS is a standardized instrument with strong psychometric properties, including high internal consistency (α = 0.93), test–retest reliability (*r* = 0.88), and concurrent validity [34]. Based on a process model of recovery, the RAS attempts to assess specific aspects of recovery with a particular focus on hope and self-determination. It is a tool for a voluntary self-reflective assessment to measure the perception of an individual’s recovery, especially following a mental health challenge [40,42]. The total score is positively associated with quality of life and empowerment, whereas it is inversely associated with symptoms [41,43].

The EQ-5D-5L scale measures the quality of life on a five-component scale, including mobility, self-care, usual activities, pain/discomfort, and anxiety/depression [36]. The descriptive system comprises five components: mobility, self-care, usual activities, pain/discomfort, and anxiety/depression [36]. Each component has five levels: no problems, slight problems, moderate problems, severe problems, and extreme problems [43]. The EQ visual analogue scale (EQ-VAS) can be used as a quantitative measure of health outcomes ranging from 0 to 100, indicating the worst imaginable and best imaginable health, respectively, and reflecting the patient’s judgment [36]. The scale is considered a reliable and valid instrument that describes health status, which can be applicable to a broad range of populations having between 0.65 and 0.91 test–retest reliability [44,45].

### 2.6. Statistical Analysis

Data analysis for this study was performed using SPSS for Mac, version 25 (IBM Corporation, New York, NY, USA) [46]. Association analysis of baseline characteristics (sociodemographic, primary diagnosis, and clinical scales) against gender groups of the participants who completed both the baseline survey (before discharge) and six weeks after they were discharged was performed using the Chi-squared test/Fisher’s exact test for the categorical variables and the ANOVA test for the continuous variables. A paired sample *t*-test was used to assess the change in the mean scores of the BRS, RAS and EQ-VAS six weeks after service users were discharged, and related data were presented using mean and standard deviation in addition to reporting on the percentage of change from baseline parameters. Chi-squared/Fisher Exact test to assess the prevalence changes from baseline to six weeks in categorical variables related to the BRS, RAS, and EQ-5D-5L. There was no imputation for missing data, and the total numbers reported represent the total responses recorded for each variable. The significance level was set for each analysis at two-tailed *p* < 0.05. 

## 3. Results

### Longitudinal Study Outcomes

A total of 334 service users were contacted for the study out of which 306 consented and signed up for the project between 8 March and 31 May 2022 and provided baseline survey data (11 declined and 17 did not meet the inclusion criteria such as not having cell phone and were below the age of 18 years). All these study participants were assigned to the control group in accordance with the stepped wedge design for the randomized trial. Overall, 144 study participants attempted the 6-week survey between 19 April 2022, and 12 July 2022; out of which 37 provided incomplete responses, 19 surveys used non-valid phone numbers (non-trackable cases), and 88 completed both baseline and six weeks and provided valid phone numbers giving an effective response rate = 88/306 = 28.8% for the six weeks survey.

Table 1 depicts the distribution of sociodemographic characteristics of the participants against their gender distribution. A total of eighty-eight study participants who completed both baseline and six-week surveys and provided a valid phone number were included in the analysis.

The majority were identified as female 55 (62.5%), 28 (31.8%) identified as male, and 5 (5.7%) identified as other gender. A considerable number of the respondents were White 60 (68.2%), had post-secondary education 43 (48.9%), were unemployed 54 (61.4%), were single 54 (61.4%), and lived in rented homes 33 (37.5%). In terms of clinical characteristics, 34 (38.6%) received a clinical diagnosis of depression/anxiety. In relation to the BRS scale scores at baseline, 55 (62.5%) had low resilience, and in terms of the EQ-5D scale, 62 (70.5%) had no problem with mobility, 72 (81.9%) had no problem with self-care, 32 (36.4%) had no problem with usual activities, 34 (38.6%) had slight pain/ discomfort while 33 (37.5%) felt slightly depressed or anxious. Overall, 68.93 (23.61), 64.80 (19.43), and 45.20 (15.87) were the mean scores for males, females, and other gender, respectively, on the EQ_VAS scale at baseline. With regards to the RAS scale, males had a mean score of 89.50 (18.36), the female mean score was 90.04 (14.17), and other gender mean score was 78.80 (15.74). No statistically significant relationship existed between the sociodemographic and clinical characteristics and gender. It should be noted that the results for the baseline data showed that most service users appeared not to have good resilience, recovery and quality of life status as measured by the respective scales before discharge from the hospital. The baseline data shows that 62.5% of service users had low resilience, 77.3% had some form of anxiety and depression, and 63.6% had pain and discomfort at discharge.

Table 2 illustrates changes in the mean scores of the clinical characteristics six weeks after hospital discharge. There was no statistically significant improvement in mean scores on the BRS, RAS and EQ-VAS from baseline to six weeks except for reliance on others which was significant (t(df) = 2.6; *p* = 0.16), an indication of deterioration from baseline (*p* > 0.05). 

Table 3 illustrates changes in the prevalence of low and normal resilience and EQ-5D categorical subscales six weeks after hospital discharge. The results suggest no statistically significant improvement in the prevalence measures associated with the RAS, BRS, and EQ-5D scales from baseline to six weeks for participants who completed both the baseline and sixth-week surveys (*p* > 0.05).

## 4. Discussion

This study compared the changes in the parameters of service users ready for discharge on the RAS, BRS, and EQ-5D scales at baseline (prior to discharge) and six weeks after discharge from acute mental health care into the community, that is, after receiving routine follow-up care but no additional interventions associated with the clinical trial. The study participants were all part of the control group of a pragmatic stepped-wedge cluster-randomized, longitudinal study currently underway in Alberta, Canada [26]. The findings of this study have shown that there was no statistically significant change in mean scores on the RAS, BRS, and EQ-VAS from baseline to six weeks (*p* > 0.05). However, a sub-scale of the recovery assessment scale, “reliance on others” was significantly lower at six weeks, an indication of deterioration from the baseline. Also, there were no statistically significant improvements in the categorical variables associated with the BRS, RAS and EQ-5D scales from baseline to six weeks for subscribers who completed both the baseline and sixth-week surveys, with about six out of ten respondents reporting low levels of resilience at both baseline and the six weeks’ time points. This lack of improvement across clinical and non-clinical domains has concerning implications for service users’ mental health and wellbeing during the critical post-discharge transition period. The findings may indicate that service users are discharged without adequate support to facilitate personal recovery trajectories, leaving them vulnerable to poor mental and physical health outcomes. The high proportion of service users meeting the threshold for low resilience is concerning, as low resilience has been linked to increased risk of anxiety, depression, and poor coping abilities [47]. Resilience can also moderate the association between anxiety and depressive symptoms [48]. Also, it is worth noting that 60% of the respondents reported having problems with usual activities. The lack of improvement on scales measuring mobility, self-care, and usual activities also raises concerns about service users’ daily functioning and abilities to reintegrate into community life after hospitalization [49]. 

The findings in this study are comparable to the outcome of descriptive research in the United States, which explored the perceptions of service users, and their families in terms of their needs, functioning, coping, and social support four weeks following discharge from inpatient treatment [17]. The outcome of this study showed that service users had residual symptoms that interfered with functioning after discharge. Persons with mental health challenges in this study identified unmet psychological needs and resources, and patient’s families found no improvements in their relatives in terms of recovery [17]. Another study conducted to assess the effect of shared decision making (SDM) in choosing community mental health rehabilitation services before discharge from mental health hospitalization revealed that participants in the intervention cohort reported greater engagement and knowledge after choosing rehabilitation services and greater service use at 6-to-12-month follow-up than those receiving standard care. It further showed that there were no differences in rehospitalization rates. The study further revealed that two significant interaction effects indicated greater improvement in personal recovery over time for the SDM cohort. The study outcome supports our findings in that those who did not receive any interventions had no improvement in their perceived personal recovery [50].

These study findings underscore the need for additional interventions to support service users’ recovery journey after discharge from inpatient mental health units. Interventions designed to help service users in the community are mostly expensive, may require face-to-face interactions, and are usually time-consuming [51,52], although some interventions, such as peer support and phone-delivered messages, as well as home visits, are likely to be cost-effective [26,43,53]. Proper treatment coordination, monitoring of the health status of service users in the community, and therapeutic and timely communication between service users and outpatient community resources staff are essential in reducing the risk of readmission and enhancing the patient’s quality of life [28]. 

Two interventions that have shown promise in providing support during the post-discharge transition are text messaging programs and peer support interventions. In a pilot study in Edmonton, Alberta, that preceded the present large-scale randomized trial utilizing peer support and supportive text messages for service users who have been discharged from inpatient mental health units, the study reported that service users who received the combined interventions of peer support and text messaging had higher recovery scores compared with those receiving treatment as usual [43]. The study recommended incorporating peer support and supportive text messages for service users discharged from acute psychiatric care [43]. Text messaging programs directly deliver cognitive behavioral therapy content and coping skills practice to service users’ mobile devices to facilitate recovery and community reintegration [26,43]. Text message delivery programs have been found to assist with the recovery of persons with mood-related problems such as anxiety and depression [54,55,56]. It is also helpful for service users with drug and addiction-related problems [56]. The outcome of a rapid review conducted by Shalaby et al. showed that texting services were reported as effective in supporting service users with psychotic disorders, substance use disorders and affective disorders [57]. The results showed high satisfaction and acceptability of the texting services for individuals with various mental health problems. Another systematic review established the relevance of text messages in managing addiction and mental health conditions such as, schizophrenia, and affective disorders [58]. 

Formal peer support interventions leverage shared experiences between previously hospitalized and newly discharged service users to provide social connection, hope, and mental health resources [26]. Peer support is a valued recovery-oriented approach [28] to persons with mental health challenges and is increasingly implemented [59,60]. Evidence indicates positive effects, better provider relationships, and increased engagement [22]. Peer support is beneficial to individuals with severe mental health problems [61]. Some key fundamental theories that underlie peer support services delivered to persons with mental health challenges are social support, social learning theory, social comparison theory, and the helper-therapy principle. The critical factor is that they are considered supportive groups with lived experience and expertise to support people coping with mental health problems [61]. Evidence shows that providing peer support to others may also benefit peer support workers by enhancing their own feelings of competence and personal value [62,63,64,65].

## 5. Limitations

The study has limitations. Firstly, the self-reported scales diminish the clinical validity of the data provided by the participants. Secondly, there was no comparison (control) group since all the respondents were part of the control group of an ongoing study. This makes it difficult to make comparisons regarding the outcome of the study. Thirdly, the relatively low response rate received in the study may limit the generalizability of the results to the whole population of discharged service users. However, a low response rate is often expected with studies using online platforms [66]. Furthermore, the researchers could not verify the responses received from the participants who provided invalid phone numbers, which could not be linked to the baseline data of these service users, thus missing a considerable portion of the received feedback. This notwithstanding, this study has provided valuable insights into the six-week trajectory for resilience, recovery, and quality of life after service users are discharged from inpatient mental health units. 

## 6. Conclusions

This study has revealed that there were no improvements in the service users on the various scales that were used to assess the level of resilience, recovery, and quality of life in the community the six weeks following discharge from acute psychiatric care, suggesting that additional support may be needed to enhance recovery. Proper treatment coordination, monitoring of the health status of service users in the community, and therapeutic and timely communication between service users and outpatient community resources staff may also enhance the resilience, recovery, and quality of life of service users discharged from the hospital. Further studies are needed into the efficacy of cost-effective and easily scalable interventions such as supportive text messaging and peer support, which have shown promise in pilot studies for providing psychological support during the post-discharge transition.

## Figures and Tables

**Table 1:** Baseline distribution of sociodemographic and clinical characteristics at baseline against resilience status.

Variables	Male, N (%) N = 28	Female, N (%) N = 55	Other, N (%) N = 5	Total, N (%) N = 88	Fisher’s Exact/ANOVA Test	*p*-Value
**Sociodemographic characteristics:**						
**Age (Years)**						
≤25	4 (14.3%)	13 (23.6%)	4 (80.0%)	21 (23.9%)	-	0.11
26–40	12 (42.9%)	21 (38.2%)	1 (20.0%)	34 (38.6%)		
41–60	7 (25.0%)	17 (30.9%)	0 (0.0%)	24 (27.3%)		
>60	5 (17.9%)	4 (7.3%)	0 (0.0%)	9 (10.2%)		
**Ethnicity**						
White	19 (67.9%)	38 (69.1%)	3 (60.0%)	60 (68.2%)	-	0.50
Indigenous	0 (0.0%)	3 (5.5%)	1 (20.0%)	4 (4.5%)		
African	3 (10.7%)	4 (7.3%)	0 (0.0%)	7 (8.0%)		
Asian	3 (10.7%)	8 (14.5%)	1 (20.0%)	12 (13.6%)		
Other	3 (10.7%)	2 (3.6%)	0 (0.0%)	5 (5.7%)		
**Educational level**						
Less than high school	4 (14.3%)	2 (3.6%)	0 (0.0%)	6 (6.8%)	-	0.41
High school	12 (42.9%)	24 (43.6%)	3 (60.0)	39 (44.3%)		
Postsecondary education	12 (42.9%)	29 (52.7%)	2 (40%)	43 (48.9%)		
**Relationship status**						
Single	20 (71.4%)	30 (54.5%)	4 (80.0%)	54 (61.4%)	-	0.45
Separated/Divorced	2 (7.1%)	9 (16.4%)	0 (0.0%)	11 (12.5%)		
Partnered/Married	6 (21.4%)	16 (29.1%)	1 (20.0%)	23 (26.1%)		
**Employment status**						
Employed	10 (35.7%)	20 (26.4%)	4 (80.0%)	34 (38.6%)	-	0.20
Unemployed	18 (64.3%)	35 (63.6%)	1 (20.0%)	54 (61.4%)		
**Housing status**						
Own home	9 (32.1%)	16 (29.1%)	0 (0.0%)	25 (28.4%)	-	0.67
Rented accommodation.	9 (32.1%)	21 (38,2%)	3 (60.0%)	33 (37.5%)		
Live with family or friends.	10 (35.7%)	18 (32.7%)	2 (40.0%)	30 (34.1%)		
**Primary Mental Health Diagnosis**						
Depression/Anxiety	7 (25.0%)	25 (45.5%)	2 (40.0%)	34 (38.6%)	-	0.25
Bipolar Disorder	5 (17.9%)	12 (21.9%)	1 (20.0%)	18 (20.5%)		
Psychosis	8 (28.6%)	5 (9.1%)	1 (20.0%)	14 (15.9%)		
Alcohol or drug use/abuse	4 (14.3%)	3 (5.5%)	0 (0.0%)	7 (8.0%)		
Other	4 (14.3%)	10 (8.2%)	1 (20.0%)	15 (17.0%)		
**Study scales and subscales:**						
**BRS scale**						
Low resilience	15 (53.6%)	35 (63.6%)	5 (100.0%)	55 (62.5%)	-	0.17
High-to-normal resilience	13 (46.4%)	20 (36.4%)	0 (0.0%)	33 (37.5%)		
**EQ-5D Scale**						
**Mobility**						
No problems walking	21 (75.0%)	37 (67.3%)	4 (80.0%)	62 (70.5%)	-	0.95
Slight problems walking	4 (14.3%)	12 (21.8%)	1 (20.0%)	17 (19.3%)		
Moderate problems walking	3 (10.7%)	4 (7.3%)	0 (0.0%)	7 (8.0%)		
Severe problems walking	0 (0.0%)	1 (1.8%)	0 (0.0%)	1 (1.1%)		
Unable to walk	0 (0.0%)	1 (1.8%)	0 (0.0%)	1 (1.1%)		
**Self-care**						
No problems washing/dressing.	24 (85.7%)	45 (81.8%)	3 (60.0%)	72 (81.9%)	-	0.40
Slight problems washing/dressing.	2 (7.1%)	7 (12.7%)	1 (20.0%)	10 (11.4%)		
Moderate problems washing/dressing.	2 (7.1%)	2 (3.6%)	1 (20.0%)	5 (5.7%)		
Severe problems washing/dressing.	0 (0.0%)	1 (1.8%)	0 (0.0%)	1 (1.1%)		
Unable to wash/dress	0 (0.0%)	0 (0.0%)	0 (0.0%)	0 (0.0%)		
**Usual activities**						
No problems doing usual activities.	9 (32.1%)	22 (40.0%)	1 (20.0%)	32 (36.4%)	-	0.82
Slight problems doing usual activities.	8 (28.6%)	11 (20.0%)	1 (20.0%)	20 (22.7%)		
Moderate problems doing usual activities.	8 (28.6%)	15 (27.3%)	3 (60.0%)	26 (29.5%)		
Severe problems doing usual activities.	3 (10.7%)	7 (12.7%)	0 (0.0%)	10 (11.4%)		
Unable to do usual activities	0 (0.0%)	0)0.0%)	0 (0.0%)	0 (0.0%)		
**Pain/discomfort**						
No pain or discomfort	12 (42.9%)	19 (34.5%)	1 (20.0%)	32 (36.4%)	-	0.92
Slight pain or discomfort	10 (35.7%)	21 (38.2%)	3 (60.0%)	34 (38.6%)		
Moderate pain or discomfort	6 (21.4%)	14 (25.5%)	1 (20.0%)	21 (23.9%)		
Severe pain or discomfort	0 (0.0%)	1 (1.8%)	0 (0.0%)	1 (1.1%)		
Extreme pain or discomfort	0 (0.0%)	0 (0.0%)	0 (0.0%)	0 (0.0%)		
**Anxiety/depression**						
Not anxious or depressed	11 (38.3%)	9 (16.4%)	0 (0.0%)	20 (22.7%)	-	0.17
Slightly anxious or depressed.	8 (28.6%)	24 (43.6%)	1 (20.0%)	33 (37.5%)		
Moderately anxious or depressed.	6 (21.4%)	15 (27.3%)	3 (60.0%)	24 (27.3%)		
Severely anxious or depressed.	2 (7.1%)	6 (10.9%)	1 (20.0%)	9 (10.2%)		
Extremely anxious or depressed	1 (3.6%)	1 (1.0%)	0 (0.0%)	2 (2.3%)		
**EQ-VAS at baseline**						
Mean score (SD)	68.93 (23.61)	64.80 (19.43)	45.20 (15.87)	-	F (2) = 2.79	0.07
**RAS scale at baseline** Mean score (SD)						
RAS total score	89.50 (18.36)	90.04 (14.17)	78.80 (15.74)	-	F (2) = 1.18	0.31
Personal confidence and hope	32.71 (8.47)	32.91 (6.33)	26.20 (5.63)	-	F (2) = 2.10	0.13
Goal and success	19.14 (4.35)	19.65 (3.58)	18.00 (4.36)	-	F (2) = 0.51	0.61
Willingness to ask for help	11.96 (1.73)	12.07 (2.18)	10.40 (3.51)	-	F (2) = 1.41	0.25
Reliance on others	16.26 (2.91)	16.84 (2.52)	17.00 (1.97)	-	F (2) = 0.51	0.60
No domination by symptoms	9.43 (3.51)	8.56 (3.16)	7.20 (3.56)	-	F (2) = 1.23	0.29

Brief resilience scale (BRS), EQ visual analogue scale (EQ-VAS); recovery assessment scale (RAS) SD: standard deviation.

**Table 2:** Change in the mean scores of the clinical characteristics six weeks after hospital discharge.

Measure	Scores, N = 88			Mean Difference (95% CI)	*p*-Value	*t* Value (df = 87)
	Baseline Score, Mean (SD)	Six-Week Score, Mean (SD)	Change from Baseline (%)			
BRS	2.75 (0.81)	2.78 (0.89)	1.09	−0.20–0.14	0.71	0.38
EQ-VAS	65.00 (21.13)	61.84 (23.29)	4.86	−2.06–8.38	0.23	1.20
RAS total	3.72 (0.66)	3.61 (0.74)	2.95	−0.01–0.23	0.87	1.7
**RAS subscales**						
Personal confidence and hope	3.61 (0.79)	3.51 (0.85)	2.77	−0.04–0.24	0.16	1.41
Goal and success	3.88 (0.77)	3.80 (0.82)	2.06	−0.07–0.23	0.30	1.05
Willingness to ask for help	3.98 (0.72)	3.84 (0.81)	3.52	−0.03–0.30	0.11	1.6
Reliance on others	4.16 (0.65)	4.00 (0.75)	3.85	−0.03–0.29	0.02	2.6
No domination by symptoms	2.92 (1.10)	2.84 (1.14)	2.74	−0.17–0.33	0.53	0.63

df: degrees of freedom. SD: standard deviation.

**Table 3:** Change in the prevalence of BRS scale and EQ-5D categorical subscales six weeks after hospital discharge.

Measures	Baseline, N (%)	Six-Weeks after Discharge, N (%)	Total	Chi-Squared/Fisher’s Exact	*p*-Value
**BRS categories**					
Normal-to-high resilience	33 (37.5%)	36 (40.9%)	69 (39.2%)	0.22	0.63
Low resilience	55 (62.5%)	52 (59.1%)	107 (60.8)		
**EQ-5D subscales**					
**Mobility:**					
No problems walking	62 (70.5%)	63 (71.6%)	125 (71.0%)	*	0.60
Slight problems walking	17 (19.3%)	16 (18.2%)	33 (18.8%)		
Moderate problems walking	7 (8.0%)	5 (5.7%)	12 (6.8%)		
Severe problems walking	1 (1.1%)	4 (4.5%)	5 (1.8%)		
Unable to walk	1 (1.1%)	0 (0.0%)	1 (0.6%)		
**Self-care:**					
No problems washing/dressing.					
Slight problems washing/dressing.	72 (81.8%)	63 (71.6%)	135 (76.7%)	3.79	0.29
Moderate problems washing/dressing.	10 (11.4%)	16 (18.2%)	26 (14.8%)		
Severe problems washing/dressing.	5 (5.7%)	5 (5.7%)	10 (5.7%)		
Unable to wash/dress	1 (1.1%)	4 (4.5%)	5 (2.8%)		
**Usual activities**					
No problems doing usual activities.	32 (36.4%)	30 (34.1%)	62 (35.2%)	4.68	0.32
Slight problems doing usual activities.	20 (22.7%)	26 (29.5%)	46 (26.1%)		
Moderate problems doing usual activities.	26 (29.5%)	20 (22.7%)	46 (26.1%)		
Severe problems doing usual activities.	10 (11.4%)	9 (10.2%)	19 (10.8%)		
Unable to do usual activities	0 (0.0%)	3 (3.4%)	3 (1.7%)		
**Pain/discomfort**					
No pain or discomfort	32 (36.4%)	31 (35.2%)	63 (35.8%)	*	0.61
Slight pain or discomfort	34 (38.6%)	64 (36.4%)	64 (36.4%)		
Moderate pain or discomfort	21 (23.9%)	43 (24.4%)	43 (24.4%)		
Severe pain or discomfort	1 (1.1%)	5 (2.8%)	5 (2.8%)		
Extreme pain or discomfort	0 (0.0%)	1 (0.6%)	1 (0.6%)		
**Anxiety/depression**					
Not anxious or depressed	20 (22.7%)	22 (25.0%)	42 (23.9%)	4.38	0.36
Slightly anxious or depressed.	33 (37.5%)	59 (33.5%)	59 (33.5%)		
Moderately anxious or depressed.	24 (27.3%)	44 (25.0%)	44 (25.0%)		
Severely anxious or depressed.	9 (10.2%)	23 (13.1%)	23 (13.1%)		
Extremely anxious or depressed	2 (2.3%)	8 (4.5%)	8 (4.5%)		

* Fisher Exact.

## Data Availability

The data presented in this study are available on request from the corresponding author. The data are not publicly available due to [privacy and ethical reasons].
